# Metallic nanoparticles in the treatment of staphylococcus infections: a scoping review

**DOI:** 10.1186/s40360-025-01067-y

**Published:** 2025-12-12

**Authors:** Christian Kelechi Ezeh, Stephen Chijioke Emencheta, Kingsley Chisom Ugwuanyi

**Affiliations:** 1 Department of Microbiology, University of Nigeria, Nsukka, Enugu State Nigeria; 2 InSight Health Group, Abuja, Nigeria; 3 Department of Pharmaceutical Microbiology and Biotechnology, University of Nigeria, Nsukka, Enugu State Nigeria; 4 Department of Public Health and Social Care, University of Derby, Derbyshire, United Kingdom

**Keywords:** *Staphylococcus aureus*, Metallic nanoparticles, Silver nanoparticles (AgNPs), Antimicrobial activity, Drug-resistant infections

## Abstract

**Background:**

Infectious diseases constitute a serious public health problem and Staphylococcus species are one of the major pathogens that cause infectious diseases. This review aims to identify metallic nanoparticles used to combat Staphylococcus infections.

**Methods:**

In this study, three (3) electronic databases (Google Scholar, PubMed, and Science Direct) were used to search for relevant literature following the Preferred Reporting Items for Systematic Reviews and Meta-Analyses extension for Scoping Reviews (PRISMA-ScR) flow chart.

**Results:**

A total of 35 studies were included in this review. Results showed that silver nanoparticles (AgNPs) were the predominant metallic nanoparticles used for combating Staphylococcus based infections. Other metallic nanoparticles include copper nanoparticles (CuNPs), selenium nanoparticles (SeNPs), aluminum nanoparticles (AlNPs), gold nanoparticles (AuNPs), Nickel nanoparticles (NiNPs), and vanadium nanoparticles (VanNPs). Also, UV-visible spectroscopy was the most frequently reported characterization method, followed by Transmission Electron Microscopy (TEM), Scanning Electron Microscopy (SEM), and X-Ray Diffraction (XRD). In the included studies, antimicrobial activities were evaluated using zone of inhibition (ZOI) and minimum inhibitory concentration (MIC) values. Reported ZOI in the various studies ranged from 4.7 mm to 32 mm, with MIC values generally between 0.25–3125 µg/mL.

**Conclusions:**

In conclusion, whereas metallic nanomaterials, particularly copper and silver nanoparticles, have promising antibacterial properties against drug-resistant strains of *Staphylococcus aureus,* more thorough and consistent reporting is obviously required in subsequent research.

## Introduction

Infectious diseases constitute one of the main causes of morbidity and mortality globally. Hence it results in a significant public health challenge. The emergence of multidrug resistant (MDR) pathogens which is usually caused by the indiscriminate use of antimicrobial agents, further exacerbates the problem associated with infectious diseases. This has directly affected public health and the economy due to higher expenditure in medical care to treat patients affected by these diseases [1]. Hence, there is a need to seek alternative antimicrobial agents to replace ineffective ones and also the need to protect the effectiveness of the existing antimicrobial agents [1].

Among pathogenic microorganisms, *Staphylococcus aureus* is one of the most clinically significant pathogens that cause infectious diseases. *S. aureus* is well adapted to various environments due to its metabolic versatility. It colonizes the skin and nasopharyngeal membranes as normal microbiota in healthy humans [2]. However, when they infiltrate bloodstream and internal tissues, they cause many harmful diseases. *S. aureus* is a significant pathogen that causes a variety of infectious diseases in both healthy people and those with underlying medical conditions. These infections includes pneumonia, bacteremia, osteomyelitis, skin infections, and endocarditis through the expression of different virulence factors [3]. Furthermore, difficulties in treating *Staphylococcus spp* infections is due to the development of resistance to different antibiotics due to the expression of antibiotic resistance genes [4] and the formation of biofilms [5]. Skin infections can lead to the development of chronic wounds, which in turn increase morbidity and clinical complications [6]. As a result, the study and development of new and the exploration of alternative antimicrobial agents to control these pathogens are considered a high priority by the World Health Organization.

Therefore, nanotechnology offers a promising approach for synthesizing functionalized nanoparticles (NPs) as alternative antimicrobial agents, especially given the urgent need for new treatments [7]. NPs can be synthesized through physical, chemical, or biological methods [8]. Among these, nanoparticles are particularly notable for their diverse biological activities, including antioxidant [9, 10], anti-inflammatory [11], anticancer [12], larvicidal [13], antimicrobial [6, 14–16], and antibiofilm [17]. NPs exhibit broad-spectrum activity, low toxicity, and compatibility with conventional drugs, making them a viable option for controlling microorganisms, including multidrug-resistant (MDR) strains [18].

Several reviews have broadly discussed the antimicrobial activity of metallic nanoparticles; however, most have focused on general bacterial pathogens rather than *S. aureus* specifically. For instance, Singh et al. [19] and Franco et al. [20] discussed general antibacterial activities of metallic nanoparticles, while Khalifa et al. [21] focused their discussion mainly on silver nanoparticles (AgNPs). Considering the relevance and impact of infections caused by *S. aureus* in public health, this scoping review provides updated insights by collating studies published that evaluate various metallic nanoparticles against *S. aureus*. By synthesizing data on antimicrobial efficacy, this review identifies current knowledge gaps and translational opportunities for developing metallic nanoparticles for staphylococcal infections treatment.

### Research objective

To identify and map the types of metallic nanoparticles currently being investigated or utilized in the treatment of *S. aureus* mediated infections.

### Search strategy

Literature search was conducted across three databases, Google Scholar, PubMed, and Science Direct for relevant articles. Search terms included “Nanomaterials, Nanoparticles, Metal-based nanomaterials, Silver nanoparticles, Copper nanoparticles, Gold nanoparticles, Zinc nanoparticles, Staphylococcus infections, *Staphylococcus aureus*, Methicillin-resistant *Staphylococcus aureus* (MRSA), and Drug-resistant bacteria. Boolean operators (OR, AND) were used to combine search terms appropriately. The search strategy was adapted to fit into the structure and indexing terms of each database. The number of records from each database was documented.

### Selection criteria

Included studies met the following criteria: (1) the study focuses on the use of metallic nanoparticles for *Staphylococcus spp* treatment, (2) they describe in vitro study, (3) the *S. aureus* is from human samples, (4) articles were written in English.

Studies were excluded if they (1) the focus was not on metallic nanoparticles, (2) described in vivo study, (3) studies that used *S. aureus* from non-human samples, and (4) studies not written in English.

### Study selection

**Fig. 1:**
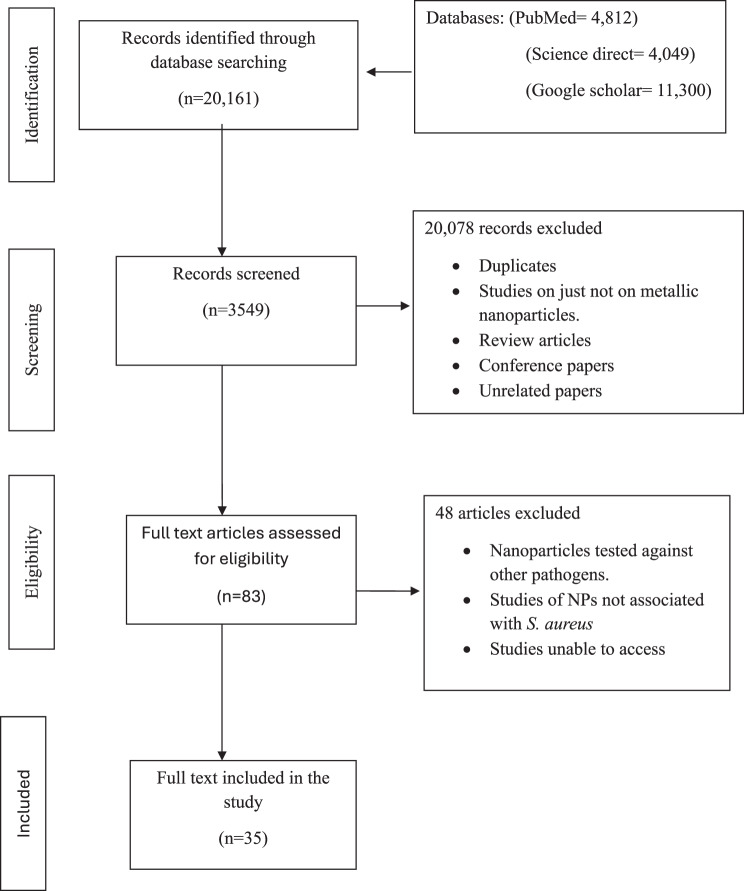
PRISMA flowchart for the selection and screening of eligible studies

Retrieved studies from the various databases were imported into the Zotero reference management software and duplicates were removed. The remaining articles were screened in two stages. First, titles and abstracts were screened for their relevance based on the inclusion criteria. Full-text screening of studies that passed the first screening was independently conducted by two reviewers CKE and SCE. Disagreements were resolved through discussion. A PRISMA-ScR flow diagram was used to document the selection process as shown in Fig. 1.

### Data extraction and synthesis

Data was extracted using a standardized form in Microsoft Excel by the two reviewers (CKE and SCE). Data extracted include Author/year, country, type of nanoparticles, method of synthesis, characterization, *S. aureus* isolate origin, type of *S. aureus*, concentration of NPs and inhibition zone diameter, minimum inhibitory concentration (MIC), and minimum bactericidal concentration (MBC).

Data extracted were synthesized thematically and narratively providing a comprehensive overview of the current evidence on the use of metallic nanoparticles for treating *Staphylococcus* infections. Tables and charts were used to present key findings, group studies by NPs types, highlight trends, and areas of concentration.

## Results

**Fig. 2:**
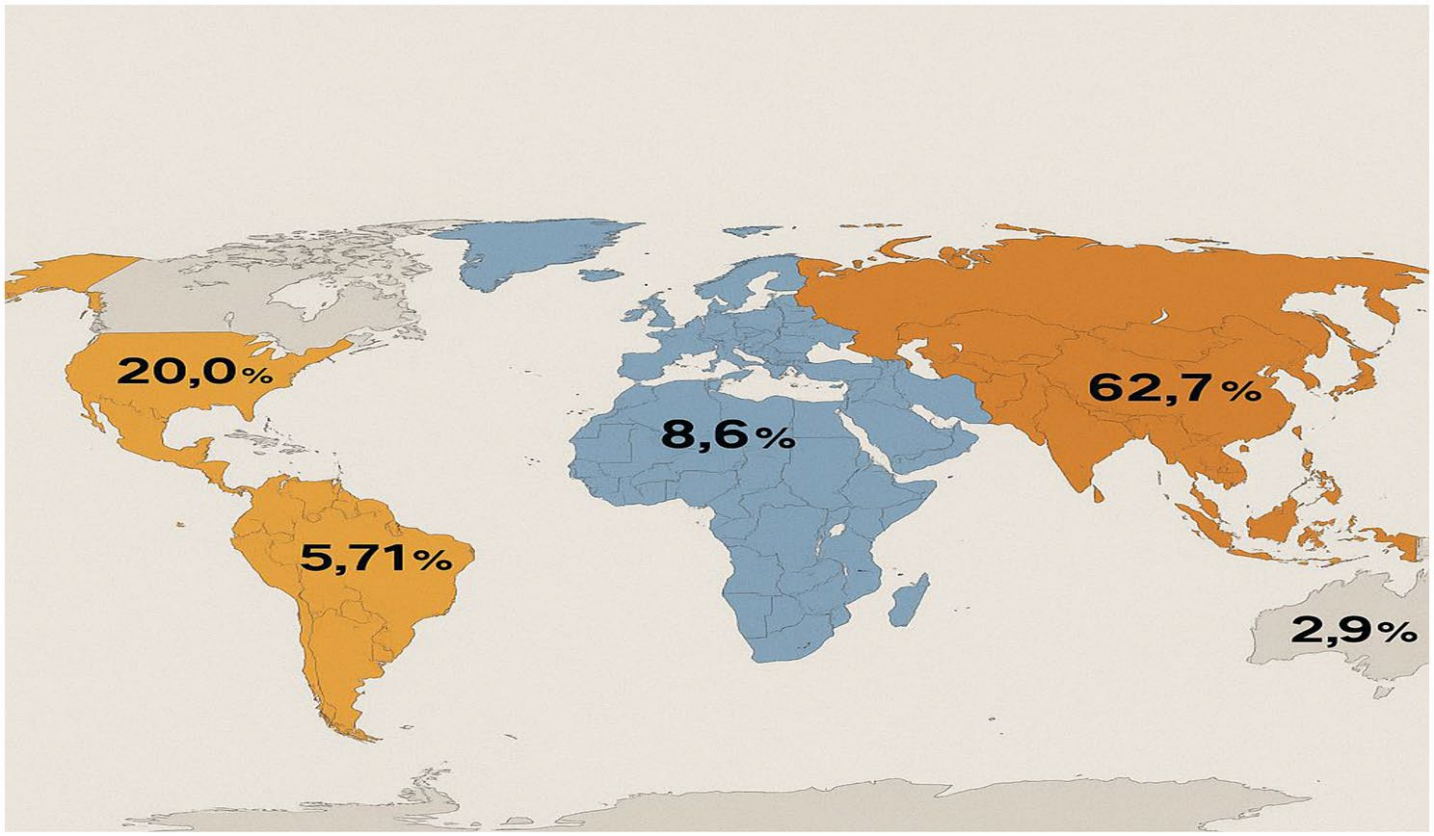
Geographical distribution of studies in diverse regions

A total of 35 identified studies that met the inclusion criteria were evaluated in this scoping review. Included studies were conducted from diverse geographic regions (Fig. 2) exploring the antimicrobial activities of metallic nanoparticles against *S. aureus*. Most of the studies tested the efficacy of metallic nanomaterials against methicillin-resistant *S. aureus* (MRSA). Most of the studies were conducted in Asia comprising 62.7% with India having the highest number of studies [9]. This is followed by Africa (20.0%), Europe (8.6%), South America (5.71%), and Australia (2.9%). This percentage showed a growing global interest in the use of nanotechnology-based solutions for combating multi-drug-resistant bacterial infections.

### Types of metallic nanoparticles and synthesis methods

Results in Table 1 showed that most of the studies used AgNPs. Other metallic nanoparticles used include Copper nanoparticles (CuNPs), Selenium nanoparticles (SeNPs), Aluminum nanoparticles (AlNPs), Gold nanoparticles (AuNPs), Nickel nanoparticles (NiNPs), and Vanadium nanoparticles (VanNPs). Also, some studies utilized a combination of metals and organic compounds such as chitosan-silver nanoparticles-graphene oxide nanohybrids, Ag-clotrimazole, mesostructured silica-Ag, and chitosan-Ag. Various methods of synthesis were reported including chemical, physical and biological methods. A total of 17 studies used biological methods (10 used plants while 7 used microorganisms), 9 studies used the chemical method, 2 studies used the physical method, 1 study used both chemical and biological methods while 6 studies did not specify (Table 1).

**Table 1:** Data of included studies

S/No	References	Country	Type of metallic nanoparticles	Method of synthesis	Characterization method used
1	[22]	Nigeria	Ag	Chemical	UV
2	[23]	Iraq	Cu	collected	-
3	[24]	Eygpt	Ag	Chemical	UV, XRD, SEM, TEM
4	[25]	India	Ag	Plant	UV
5	[26]	Pakistan	Ag	Bacteria	UV, TEM
6	[27]	Malaysia	Ag	Plant	UV, TEM, SEM, FTIR
7	[28]	Eygpt	Ag	cyanobacteria (Desertifilum sp.	UV, SEM
8	[29]	Iran	Ag	cyanobacteria	UV, Zeta potential, DLS, TEM, FTIR
9	[30]	Eygpt	Ag	Plant	XRD, FTIR, UV, TEM, SEM, EDX
10	[31]	India	Ag	Fungi	UV, FTIR, SEM
11	[32]	Romania	chitosan–silver nanoparticles-graphene oxide nanohybrids	chemical	UV, TEM, XPS, Zeta potential
12	[33]	South Africa	Ag -cltrimazole		Differential scanning calorimtry, XRD
13	[34]	Taiwan	Mesostructure silica-Ag		UV, FTIR, TEM, XRD
14	[35]	India	Ag	plant	UV, FTIR, XRD, TEM, EDX
15	[36]	India	Ag	Bacteria	UV, AFM
16	[37]	Korea	Van, Ag, Au	Chemical	HR-XRD, HR-TEM, FT,AFM, UV
17	[38]	Brazil	Ag	Chemical	SEM
18	[39]	India	Al2O2	Bought	SEM, XRD,
19	[40]	Ukraine	Chitosan-AgNPs	Plant	XRD, FTIR, UV TEM,
20	[41]	Eygpt	Ag	Plant	UV, TEM, EDX
21	[42]	Eygpt	Ag	chemical	
22	[43]	Australia	Selenium	Chemical	TEM, XPS, SEM
23	[44]	Columbia	Ag	Chemical	UV, TEM, DLS
24	[45]	India	NiONps	plant	UV, FTIR, XRD, EDX,SEM
25	[46]	Iran	Ag	Physical	XRD, SEM
26	[47]	India	Ag	Plant	UV, DLS, TEM, SEM, XRD
27	[48]	Saudi Arabia	Ag	Bacteria	UV, TEM, FTIR
28	[9]	Iran	Ag	Chemical/plant	UV, DLS, FTIR, XRD
29	[49]	Iran	Ag	Plant	UV, XRD, FTIR,
30	[50]	Taiwan	Ag	Chemical	UV, Zeta, TEM, XRD
31	[51]	Netherland	Ag	Physical	SEM, DLS, TEM, FTIR
32	[52]	India	Ag	Chemical	UV, TEM, DLS, Zeta
33	[53]	Singapore	Ag	Plant	UV, FTIR, EDX, TEM, SEM
34	[54]	China	Ag		TEM, XRD, FTIR, DLS
35	[55]	India	Ag, Au	Fungi	XRD, HR-TEM, FTIR, EDX, UV

Key: UV = Ultraviolet Spectroscopy, XRD = X-ray Diffraction, SEM = Scanning Electron Microscopy, TEM = Transmission Electron Microscopy, FTIR = Fourier Transform Infrared Spectroscopy, DLS = Dynamic Light Scattering, EDX = Energy Dispersive X-ray Spectroscopy, XPS = X-ray Photoelectron Spectroscopy, AFM = Atomic Force Microscopy, HR-XRD = High-Resolution X-ray Diffraction, and HR-TEM = High-Resolution Transmission Electron Microscopy.

### Characterization methods

Characterization of metallic nanoparticles was inconsistently reportedly across studies. Characteristically, UV-visible spectroscopy was the most frequently reported method, followed by Transmission Electron Microscopy (TEM), Scanning Electron Microscopy (SEM), and X-Ray Diffraction (XRD). These methods provided insights into nanoparticle size, shape (spherical), and optical properties. Other characterization methods include Fourier transform infrared spectroscopy (FTIR), Zeta potential, Energy Dispersive X-ray Spectroscopy (EDX), X-ray photoelectron spectroscopy (XPS), Atomic Force Microscopy (AFM), and Dynamic Light Scattering (DLS) (Table 1).

### Origin of staphylococcus aureus isolates

*S. aureus* were isolated from a range of clinical samples such as urine, blood, wounds, sputum, and high vaginal swabs (HVS). The predominant bacterial targets in most of the studies were MRSA, with some studies also addressing methicillin-sensitive *S. aureus* (MSSA) and vancomycin resistant *S. aureus* (VRSA) strains. The high emphasis on MRSA could be attributed to its resistance to multiple antibiotics especially the first line antibiotics (beta-lactam antibiotics) and its ability to cause life threatening infections (Table 2).

**Table 2:** Isolates properties and antibacterial activities of nanoparticles

Metallic nanoparticles	Isolate origin	Type of *S. aureus*	Concentration (μg/mL)	Zone of inhibition (mm)	MIC (μg/mL)	MBC (μg/mL)	References
Ag	Mix (blood, urine, HVS, wound, ear, semen)	MRSA MSSA	2.5 - 10	MRSA = 4.7 MSSA = 4.9	5		[22]
Cu	Wound	MRSA	150	18	75–150	75–150	[23]
Ag	Sputum and blood	MRSA VRSA	90	23			[24]
Ag	Wound	MRSA	100	32			[25]
Ag	clinical	MRSA	80	18			[26]
Ag	clinical	MRSA	1.25 - 10	16.67	1.25	2.5	[27]
Ag	clinical	MRSA	1540	23	1.2	1,500	[28]
Ag	clinical	MRSA			14.9	14.9	[29]
Ag	sputum	MRSA	75–100	19			[30]
Ag	clinical	MSSA	Disc	20			[31]
chitosan–silver nanoparticles-graphene oxide nanohybrids	clinical	MRSA			1.09	1.35	[32]
Ag -cltrimazole	clinical	MRSA MSSA			MRSA = 15.62, MSSA = 9.76		[33]
Mesostructure silica-Ag	clinical	MRSA			2,500–5000		[34]
Ag	wound	MSSA		80			[35]
Ag	clinical	MRSA MSSA	20	17			[36]
Van, Ag, Au	clinical	MRSA			45.3–90.6		[37]
Ag	clinical	MRSA MSSA			1.95	3.91	[38]
Al2O2	clinical	MRSA MSSA			1,700	3,400	[39]
Chitosan-AgNPs	clinical	MRSA			6		[40]
Ag	clinical	MRSA			3.13		[41]
Ag	clinical	MRSA			4	8–16	[42]
Selenium	clinical	MRSA			0.5		[43]
Ag	clinical	MRSA			0.25	1	[44]
NiONps	Pus, urine, surgery, wound, and blood	MRSA MSSA	1,000	MRSA = 14 MSSA = 15	800	1.6	[45]
Ag	clinical	MRSA	20,000	24.2	0.015	70	[46]
Ag	clinical	MSSA	32	16	8	16	[47]
Ag	clinical	MSSA			230		[48]
Ag	clinical	MSSA			1,280	2,400	[9]
Ag	clinical	MSSA			20	60	[49]
Ag	clinical	MSSA			48.16		[50]
Ag		MSSA			16		[51]
Ag	clinical	MRSA MSSA			MRSA = 19.13 MSSA = 9.7		[52]
Ag	clinical	MSSA	1,000	18.3	90	90	[53]
Ag	clinical	MRSA MSSA	500	14	10,000	2,000	[54]
Ag, Au	clinical	MRSA	1,000	16.15	3125		[55]

### Antibacterial efficacy

In the included studies, antimicrobial activities were evaluated using zone of inhibition (ZOI), minimum inhibitory concentration (MIC), and minimum bactericidal concentration (MBC) values as shown in Table 2. The reported ZOI in the various studies ranged from 4.7 mm to 32 mm. However, not all studies reported detailed quantitative data. Notably, the study by Kalugendo and Kousalya [25] and Saleem et al. [26] demonstrated substantial ZOIs of 18 mm and 32 mm respectively, which is indicative of the strong antibacterial activity of the synthesized metallic nanoparticles. The highest IZD reported was against MRSA at 100 µg/mL.

MIC of the studies ranged from 0.25–3125 µg/mL. The best MIC value (0.25 µg/mL) was reported against MRSA and it was recorded by chemically synthesized AgNPs [44]. Also, Balakumaran [55] reported that Fungi synthesized AuNPs had an MIC of 3125 μg/mL against MRSA. AlNPs had an MIC of 1700 μg/mL against both MRSA and MSSA, Chitosan-AgNPs (6 μg/mL), SeNPs (0.5 μg/mL), and NiNPs had an MIC of o.8 μg/mL. MBC as reported in the studies [15] ranged from 1.35–3400 μg/mL. The lowest (1.35 µg/mL) MBC was recorded by chitosan–silver nanoparticles-graphene oxide nanohybrids [32] while the highest (3,400 µg/mL) MBC was recorded by AlNPs [39].

In summary, across the included studies, AgNPs were the most frequently evaluated and demonstrated the strongest antibacterial effects against both MRSA and MSSA isolates, with zones of inhibition (ZOI) ranging from 4.7–32 mm and MIC values between 0.015–1280 μg/mL. CuNPs exhibited moderate antibacterial activity (ZOI = 18 mm; MIC = 75–150 μg/mL), while SeNPs showed high potency at very low concentrations (MIC = 0.5 μg/mL). NiONPs demonstrated ZOI values of 14–15 mm and MIC values around 800 μg/mL. Other hybrid or composite NPs, such as chitosan–silver or Ag–Au formulations, also showed enhanced activity, with MIC values as low as 1.09 μg/mL.

### Overall trends and gaps

- Among all the metallic nanoparticles, AgNPs are the most studied NPs and effective against *S. aureus* strains.
- As expected, more than 80% of studies used UV spectroscopy as the first characterization tool, however, fewer studies reported detailed structural or functional analysis using SEM, TEM, and XRD).
- About 48.6% of studies used either plant or bacterial for metallic nanoparticles synthesis, showing a growing shift toward biological synthesis.
- Despite promising zone of inhibition (ZOI) results, 80% reported MICs and just 45.7% included MBCs, underscoring a need for more comprehensive pharmacodynamic data.
- Inconsistent reports, such as missing synthesis methods and incomplete characterization methods, and results in some studies, limited the ability to perform a direct studies comparison.
- Toxicity, cytocompatibility, and clinical translation were rarely addressed in the reviewed studies.

## Discussion

Findings from this scoping review showed a significant global interest in the antimicrobial activities of metallic nanoparticles, especially against multidrug-resistant strains of *S. aureus* such as MRSA and VRSA. The review did not include a meta-analysis, as the objective is to map the available evidence rather than assess pooled effect sizes. The studies reviewed were conducted in diverse geographical regions including Asia, Africa, South America, Europe, and Australia, which underscores the evolving role of nanomedicine in combating the growing global health concern of antimicrobial resistance (AMR). This wide range geographic distribution underscores the global recognition of metallic nanoparticles as a promising solution for combating multidrug-resistant bacterial infections [56]. This geographic information was verified from each study’s methods section to ensure accuracy. Inclusion of study location demonstrates the broad interest and adoption of nanoparticle research across multiple continents, reflecting the global urgency to address antimicrobial resistance.

AgNPs were the most synthesized, with CuNPs, AuNPs, AlNPs, SeNPs, and NiNPs being explored in a single study each. The high preference for AgNPs synthesis is likely due to their unique properties suitable for biological applications such as antimicrobial, antioxidant, and catalytic properties, high surface area-to-volume ratio, and tunable size and shape. They also exhibit strong surface plasmon resonance, biocompatibility, and stability, making them effective for drug delivery, biosensing, wound healing, and antimicrobial formulations [57].

The review also reveals that various synthesis methods were employed across the studies, including chemical, biological (plant- or bacteria-mediated), and physical approaches. While chemical synthesis methods are used for synthesis in 9 studies, the emergence of biological or green (use of plants or microorganisms) synthesis indicates a growing trend towards environmentally sustainable fabrication of nanoparticles. Biological synthesis methods utilizing natural resources (plants and microorganisms), offer advantages including reduced toxicity, cost effectiveness, and environmentally friendly, aligning with the broader push for eco-friendly technologies [58, 59]. Biologically mediated NPs may carry capping biomolecules that modulate interactions with bacterial membranes, sometimes enhancing or reducing efficacy depending on the system [58]. In contrast, studies have reported that chemical synthesis methods, although widely adopted, can result in the introduction of toxic by-products which can hinder the biocompatibility of the NPs [60].

Characteristically, UV-visible spectroscopy was the most used technique across studies, which provides insights into the optical properties of the synthesized nanoparticles. Other characterization techniques, such as SEM, TEM, and XRD, were used to evaluate the size, shape (predominantly spherical), and structural properties of the nanoparticles. These techniques are critical for ensuring the uniformity and quality of the synthesized nanoparticles, which are significant factors influencing their antimicrobial properties. However, inconsistent nanoparticle characterization hinders the reproducibility of results and poses challenges for comparing the antibacterial potential across studies. For future studies, standardized reporting of key parameters, including particle size distribution, zeta potential, shape, polydispersity index, and synthesis source are required, as these factors critically influence antimicrobial activity.

The origin of the *S. aureus* isolates in the included studies is from clinical samples ranging from urine, blood, sputum, wounds, and high vaginal swabs (HVS), with MRSA being the predominant target pathogen. Some studies also investigated MSSA and VRSA strains, reinforcing the focus on multidrug-resistant bacteria, which are increasingly difficult to treat using conventional antibiotics. This wide range of strains of *S. aureus* reflects the diverse challenges posed by antimicrobial resistance and the need for alternative antimicrobial agents. This can be echoed in a study that emphasized that antimicrobial stewardship programs can drastically reduce the rate of resistance toward antibiotics [61].

The antimicrobial activity of metallic nanoparticles has been extensively documented across several studies, with ZOI and MIC values being the primary quantitative measures [62, 63]. In the included studies, ZOIs ranged from 4.7 mm to 32 mm, and MICs ranged from 2.5 to 2000 µg/mL, showcasing a significant variation depending on nanoparticle type, synthesis method, and target organism.

Notably, Kalugendo and Kousalya [25] and Saleem et al. [26] reported significant ZOIs of 18 mm and 32 mm, respectively. It has been reported that ZOIs greater than 15 mm generally indicate potent antibacterial activity [64], advocating the view that the fabricated metallic nanoparticles possess strong bactericidal activities. Saleem et al., (2020) reported ZOI of 32 mm against MRSA particularly underscoring the superior potency of their NPs formulation. The results from various studies showed that there is a significant variation in the effectiveness and efficacy of the formulated NPs, necessitating further characterization and optimization. In addition, these findings support the continued advancement of research and possible clinical translation of NPs- based antimicrobials.

MIC values give a more sensitive measure of antimicrobial potential than ZOI, because they give the minimal concentration required to inhibit visible microbial growth [65]. In the included studies evaluated, MICs ranged from 0.25–3125 µg/mL. Ortiz et al. [44] reported the best MIC value of 0.25 µg/mL against MRSA using chemically synthesized silver nanoparticles (AgNPs), highlighting the high potency of AgNPs against resistant bacterial strains. The superior antimicrobial activity of AgNPs can be attributed to multiple mechanisms: silver ions (Ag^+^) bind to thiol (–SH) groups in bacterial enzymes, inactivating key metabolic proteins; AgNPs disrupt cell membranes and increase permeability; they generate reactive oxygen species (ROS) that damage proteins, lipids, and DNA; and they interfere with DNA replication and transcription [66]. In addition, physicochemical factors such as particle size, shape, surface charge, and capping agents also modulate their efficacy by influencing ion release, membrane interaction, and ROS generation [67]. Composite or hybrid systems, such as chitosan–graphene oxide–AgNPs, reveal synergistic antimicrobial effects. Chitosan’s polycationic nature promotes binding to negatively charged bacterial membranes, the matrix stabilizes nanoparticles to prevent aggregation, and the embedded metal nanoparticles release ions and ROS for a multi-mode bactericidal effect. Such synergy often results in lower MIC and MBC values than single-component nanoparticles [68].

In contrast, Balakumaran et al. [55] reported a significantly higher MIC of 3125 μg/mL using fungi-synthesized AuNPs against MRSA. Such high MIC values suggest a comparatively weaker antibacterial potency, which could be attributed to differences in nanoparticle size, surface charge, morphology, or the presence of capping biomolecules from fungal synthesis, which may hinder effective bacterial interaction [60].

AlNPs also exhibited relatively high MIC values (1,700 μg/mL) against MRSA and MSSA, highlighting limited antibacterial potency compared to AgNPs. Chitosan-coated AgNPs, however, exhibited a much lower MIC of 6 μg/mL, reflecting the synergistic effect of chitosan and AgNPs. Chitosan itself exhibit antimicrobial activities due to its polycationic nature, which facilitates interaction with negatively charged microbial cell membranes [69].

Other notable results include SeNPs with an MIC of 0.5 μg/mL and NiNPs with an MIC of 0.8 μg/mL. These findings suggest that less traditionally explored metallic nanoparticles (SeNPs, NiNPs) may hold promise for future antimicrobial applications. From the MIC results, it can be suggested that synthesis methods significantly influence NPs efficacy. Therefore, optimizing NPs properties is essential for developing an effective antimicrobial agent.

MBC results varied from 1.35 to 3400 μg/mL across the studies. Marta et al. [32] demonstrated the strong bactericidal effect that may be achieved by combining numerous nanomaterials by reporting the lowest MBC (1.35 µg/mL) with a chitosan–graphene oxide–silver nanoparticle nanohybrid. This suggests that synergistic antibacterial effects when two or more nanomaterials are combined. The comparatively poor bactericidal effectiveness of AlNPs was further confirmed by the fact that AlNPs had the highest MBC (3,400 µg/ml [39]. Findings showed that NPs composition aids synergistic activities which are critical in determining bactericidal strength. The implications is that this hybrid offers greater potential for fabricating new therapeutic agents that can kill pathogenic bacteria at a very low dose and improve treatment outcomes.

Overall, the findings showed that a variety of parameters, such as the synthesis process, nanoparticles composition, particle size, surface changes, and the particular microbial strain targeted, affect the antibacterial activity of NPs. As one of the most effective NPs type for antibacterial applications, AgNPs continuously beat NPs types [70]. Moving forward, rigorous characterization, toxicity evaluation, and clinical validation are essential to translating these promising laboratory findings into safe and effective therapies.

## Conclusion

In conclusion, whereas metallic nanoparticles, particularly CuNPs and AgNPs, have promising antibacterial properties against drug-resistant strains of *S. aureus*, more thorough and consistent reporting is obviously required in subsequent research. This includes investigating the toxicity and modes of action of nanoparticles, improving the definition of MIC and MBC values, and using thorough characterization techniques. Future studies should adopt standardized characterization protocols, evaluate cytotoxicity and pharmacokinetics, and explore synergistic hybrid formulations for clinical applications. Additionally, advancement in nanotechnology-based solutions could open up new possibilities for the creation of potent remedies for bacterial infections that are resistant to antibiotics, as the globe struggles with antimicrobial resistance.

## Data Availability

Available on demand
